# Percutaneous tracheostomy in critically ill patients: 24 months experience at a tertiary care hospital in United Arab Emirates

**DOI:** 10.4103/1817-1737.58956

**Published:** 2010

**Authors:** Raees Ahmed, Sherif R. Rady, Javed Iqbal Mohammad Siddique, Mobeen Iqbal

**Affiliations:** *Rashid Hospital Trauma Center, Medical ICU, PO Box 4545, Dubai-UAE*; 1 *Mafraq Hospital, Medical ICU, Abu Dhabi-UAE*; 2 *Shifa College of Medicine/Shifa International Hospital, Islamabad, Pakistan*

**Keywords:** Complications, percutaneous tracheostomy (PCT), safety

## Abstract

**OBJECTIVE:**

We assessed the safety and complications related to percutaneous tracheostomy (PCT) without bronchoscopic guidance in our intensive care unit (ICU).

**METHODS:**

The prospective data over a period of 24 months were collected for patients who underwent PCT. Major, minor and long-term complications were recorded. The parameters recorded were: age, gender, Glasgow Coma Scale (GCS) score on the day of tracheostomy, acute physiology and chronic health evaluation II (APACHE) score, and predicted mortality based on score on admission and on the day of procedure, number of days on ventilator before and after the procedure, total number of days in the hospital before the final outcome, number of successful decannulations and mortality. The patients were stratified in two groups of survivors and nonsurvivors.

**RESULTS:**

A total of 117 patients underwent PCT. Overall mean GCS and APACHE-II scores before PCT were 7 ± 3 and 16 ± 5, respectively. The only significant difference was APACHE-II score and the predicted mortality based on APACHE-II score on the day of PCT, which was higher amongst the nonsurvivors (*P* = 0.008 and *P* = 0.006). All 57 (49%) survivors were successfully decannulated with mean post tracheostomy days of 24 ± 15. The major complication observed was three episodes of major bleeding. Only six patients had an episode of desaturation during the procedure and there were three episode of accidental puncturing of endotracheal (ET) tube pressure cuff. During subsequent follow-up in hospital, six patients developed stomal cellulitis.

**CONCLUSIONS:**

PCT without bronchoscopic guidance can be performed safely by carefully selecting patients and having an experienced team High APACHE score on the day of procedure may lead to poor outcome.

Tracheostomy is one of the most commonly performed surgical procedures in critically ill patients requiring long-term mechanical ventilation.[1–3] Percutaneous tracheostomy (PCT) is now a well established technique used in the critical care setting. The evidence that PCT is superior to standard surgical tracheostomy (ST) is debatable. Investigators who endorse PCT as the preferred technique of airway access maintain that PCT is cost-effective, safe, fast, and easy to perform. However, certain PCT steps, such as endotracheal (ET) tube replacement and blind formation of tracheal stoma, can potentially cause serious perioperative complications.

PCT has been utilized in the Middle East (mainly in Saudi Arabia and Bahrain)[4,5] but the data about the safety and complications without bronchoscopic guidance are lacking. The aim of the present study was to look at the safety and complications of PCT without bronchoscopic guidance in our hospital. Moreover we intended to study any possible differences in survivors and nonsurvivors who underwent PCT related to duration of hospital stay, all cause mortality and search for high-risk group by using physiologic parameters.

## Methods

Intensive care unit (ICU) at Mafraq hospital, the largest level II trauma center of the city, is a 12 bed combined surgical and medical ICU. There are on an average 550 annual admissions. There is round the clock on site intensivist coverage in ICU. Nearly 60% of the admissions belong to trauma category, which include road traffic accidents and work related injuries.

We collected data beginning from 7/2005 prospectively for all patients, who underwent tracheostomy in the ICU till 6/2007. The study was approved by the Ethical and Research Committee of the hospital and the requirement of the consent was waived, as no new drug or intervention was involved in the study.

The preferred procedure was bedside PCT. PCTs were performed after obtaining the informed consent by the next to kin. The procedure was performed without direct fiberoptic bronchoscopic inspection. All patients had normal platelets count and coagulation profile before the procedure. The procedure was performed or supervised by the same senior Intensivist, with the surgical coverage from otolaryngologists and experienced anesthesiologist who was responsible for the airway management. The procedure was performed by two operators, using the technique described by Griggs *et al*. and marketed by Portex (Portex; Hythe, Kent, UK) using a dilating forceps introduced over the guide wire to form the stoma.

Immediately after the procedure, the placement of tracheostomy tube, was confirmed by adequate bilateral air entry into the lungs by chest auscultation, end tidal CO_2_ (ETCO_2_), tidal volume on ventilator and immediate post procedure chest X-ray.

The complications recorded during the procedure were classified into major, minor and long-term categories. Major complications included excessive bleeding requiring surgical intervention or blood transfusion, formation of false passage during dilatation, posterior tracheal wall perforation, post procedure sub-cutaneous emphysema, pneumothorax and death within 24 hours of the procedure. Minor complications were defined as hemodynamic instability, episodes of desaturation during the procedure, accidental extubation, puncturing of ET pressure cuff and deterioration of respiratory parameters in 24 hours post procedure. All patients were followed till their final outcome, which was hospital discharge, transfer to other facility or death, to document long term complications like stomal infection, subglottic stenosis, tracheal stenosis, tracheoesophageal or tracheocutaneous fistula.

The patients were stratified in two groups of survivors and nonsurvivors. The data collected were: age, gender, Glasgow Coma Scale (GCS) score on the day of tracheostomy, Comorbidities on admission and on the day of procedure, acute physiology and chronic health evaluation II (APACHE) score and predicted mortality based on admission and on the day of procedure, number of days on ventilator before and after the procedure, total number of days in the hospital before the final outcome, number of successful decannulations and mortality.

The data were analyzed by using statistical package SPSS (version 15, Chicago, IL, USA). Students T test was used for comparing means of the numeric data.

## Results

There were a total of 124 patients, who underwent tracheostomy in ICU during the 24 months of study period. Seven patients electively had surgical tracheostomies done in operating room due to anatomical limitation and inadequate anatomical landmarks for the bedside PCT. Patients who underwent surgical tracheostomies were excluded from the review.

There were 99 (85%) males and 18 (15%) females. Mean age was 46 ± 17 years for males and 66 ± 24 years for females. There were 57 (49%) patients with road traffic accidents resulting in polytrauma and head injury, while 60 (51%) patients had medical reasons for admission. Among the medical group of patients 18 (30%) were admitted with the diagnosis of sepsis, 27 (45%) with hemorrhagic or ischemic cerebrovascular accident, 12 (20%) with cardiopulmonary dysfunction and 3 (5%) with acute poisoning.

Most of the polytrauma patients were young adults, but the most common co morbidities among both medical and polytrauma group was Hypertension (62%) followed by diabetes mellitus (47%) and known coronary artery disease (23%).

Overall mean GCS and APACHE-II scores before PCT were 7 ± 3 and 16 ± 5, respectively.

Table 1 shows age, sex, APACHE-II and GCS scores of survivors and nonsurvivors. The only significant difference was APACHE-II score and the Predicted mortality based on APACHE-II score on the day of PCT, which was higher amongst the nonsurvivors (*P* = 0.008 and *P* = 0.006). 60(51%) patients with tracheostomy died, out of them 24 (40%) had road traffic accident, 18 (30%) had neurological problems and 18 (30%) were admitted for cardiopulmonary dysfunction. All patients 57(49%) who survived were successfully decannulated with mean post tracheostomy days of 24 ± 15.

**Table 1:** Comparison of survivors and nonsurvivors

Variables	Survivor (n = 57)	Nonsurvivor (n = 60)
Age	43.8 ± 18	54.2 ± 20
Male n (%)	57 (100)	42 (70)
Days on ventilator before tracheostomy	9.7 ± 3.8	12.1 ± 6.9
APACHE-II on admission	17.9 ± 6.4	19.3 ± 6.7
Predicted mortality by APACHE-II on admission	38.16 ± 6.4	42.32 ± 5.3
APACHE-II on the day of tracheostomy	14.1 ± 5.5	18.3 ± 3.7*
Predicted mortality by APACHE-II on the day of tracheostomy	25 ± 5.2	31.2 ± 6.2†
GCS on the day of tracheostomy	7.1 ± 3.2	7.5 ± 3.6
Days on ventilator after tracheostomy	8.7 ± 14.6	27.9 ± 43.2
Total hospital stay before final outcome	25.3 ± 23.4	41.1 ± 42.7

* *P* value = 0.008;

† *P* value = 0.006; APACHE-II = Acute physiology and chronic health evaluation; GCS = Glasgow coma scale

The complications are mentioned in Table 2. The major complication observed during 24 months of study were three episode of major bleeding during PCT, which required termination of procedure and urgent transfer of the patient to the operating room for hemostasis and insertion of ST.

**Table 2:** Complications for patients undergoing percutaneous tracheostomy

Variables	n (%)
PCT-associated bleeding	3 (2.5)
Desaturation during PCT	6 (5)
Perforation of ET pressure cuff	3 (2.5)
Posterior wall perforation	0
Stomal cellulitis/Infection	6 (5)
False passage during tube insertion	0
Death within 24 hours of PCT	0
Pneumothorax/Subcutaneous emphysema	0
Tracheal stenosis/Subglottic stenosis	0
Tracheoesophageal fistula/Tracheocutaneous fistula	0

TPC:Percutaneous Tracheostomy

Six patients who had episodes of desaturation during the procedure, with minimum oxygen saturation as low as 88%, were immediately managed by adjusting the ET tube and tracheal suctioning. There were three episode of accidental puncturing of ET tube pressure cuff, while locating the trachea, this complication was recognized immediately by low minute ventilation and rise in ETCO_2_, the procedure was temporarily stopped and new ET tube was inserted. No other major or minor complication was recognized.

During subsequent follow-up in hospital, six patients developed stomal cellulitis, which responded well to local and systemic antibiotics.

## Discussion

This is the first ever report on PCT from any institute in United Arab Emirates. Our prospective study reconfirms the safety of PCT especially without bronchoscopic guidance. The placement of the tracheal cannula using PCT was successful in 97.4% of patients. We attribute our low complication rates to our careful selection of patients and an experienced team performing the procedure.

The technique for ST was formalized in 1909 by Chevalier Jackson Sr.[6] In 1955, Shelden and colleagues reported the first attempt to perform PCT.[7] In 1985, Ciaglia and colleagues described the percutaneous dilatational tracheostomy, a method based on needle guide wire airway access followed by serial dilations with sequentially larger dilators.[8] In 1990, Griggs and colleagues reported the guide wire dilating forceps (GWDF) method. This method is based on a forceps without a cutting edge on the tip of the instrument.[9]

There are several studies where standard ST has been compared with PCT. The finding of complications was mixed, but the most of the studies found PCT to be cost effective.[10–12] We did not do the cost analysis of the two procedures in our setting but we anticipate potentially more cost savings with our PCT (compared with bronchoscopic guidance) where additional cost of bronchoscopy operator and trained bronchoscopy nurse can be excluded. In a meta-analysis published by Dulguerov and colleagues (38 studies dealing with ST and 27 dealing with PCT, published between 1960 and 1996) concluded that PCT was associated with a higher prevalence of perioperative complications including perioperative deaths and cardiorespiratory arrests.[10] In contrast, Freeman and colleagues concluded from a meta-analysis of five prospective controlled studies involving 236 patients that PCT was easier to perform, produced fewer overall postoperative complications, had shorter operative times, less postoperative and perioperative bleeding, and fewer postoperative stomal infections compared to ST.[11] In a recent meta-analysis comparing surgical and PCT, Higgins reviewed 368 abstracts, 15 prospective, randomized-controlled trials involving nearly 1,000 patients, and concluded that there is no clear difference but a trend toward fewer complications with PCT. Moreover PCT was found to be cost-effective when compared with ST.[12]

The most common indication for PCT in our patient population was poor neurologic function and low GCS, either due to head trauma and ischemic/hemorrhagic cereberovascular accident, reflecting the common practice of performing tracheostomies worldwide.[13]

In 1989, the consensus conference on artificial airways in patients receiving mechanical ventilation issued the following statement: “If the need for an artificial airway is anticipated to be greater than 21 days, a tracheotomy is preferred”.[14]

We didn't risk stratify patients to plan the timing of tracheostomies in our cohort. However, there are several small studies in the literature that supports early tracheostomies in a certain subsets of patients.[15–17]

The highlighting feature of our study is significantly low rates in all three categories of complications in spite of using blind insertion technique. Most of the published studies with low complication rates had the procedure done under direct guidance by using fiber optic bronchoscopy.

Polderman and colleagues have compared complication rates for PCT with and without bronchoscopic guidance and found that the overall rate of moderately severe complications (i.e., bleeding requiring the red blood cells (RBCs) transfusion, deterioration in respiratory function lasting >48 h, malpositioning of the cannula, stomal infection or tracheal stenosis, coagulopathy requiring the transfusion of Fresh frozen plasma (FFP) or platelets) were 4% for patients who had PCT without bronchoscopic guidance, and 3% for patients who had PCT under bronchoscopic guidance. Transient deterioration in respiratory parameters (for >36 h) was seen in 2.3% without bronchoscopic guidance and in 1.5% of patients when bronchoscopic guidance was used.[18]

Bliznikas in their systematic review of several studies recruiting 3520 patients, who underwent PCT with bronchoscopic guidance reported a successful decannulation rate of 36%, mortality of 0.4% within 24 hours of the procedure, 2.4% with mild to moderate bleeding complications, 0.8% with false passage, 0.9% with stomal infection and 1.9% developed tracheal stenosis.[19]

Kost published their 13 years experience of PCT, there were 500 critically ill patients who required PCT, and the complication rate was 9.2%, half of them with minor complications. The two most common complications were oxygen desaturation in 14 cases and bleeding in 12 cases.[20]

The strength of our study is prospective data collection, complete follow-up of all patients till their final outcome and internationally acceptable complication rates. We attribute our low complication rates to our careful selection of patients (normal platelets and coagulation profile) and insertion procedure, in which PCT was performed by an experienced team of a senior Intensivist and an otolaryngologist providing the option of converting the procedure to a classic ST if necessary. The airway was guarded by an anesthesiologist with experience in (difficult) airway management. We think that these logistical factors contributed to the low incidence of complications and the absence of procedure related fatalities in our patients. Although in our study three patient required the conversion of PCT to ST, the presence of an otolaryngologist may have helped to decrease the anxiety surrounding the procedure, and in this way contributed to optimal circumstances. The credit of low rates of long term complications goes to the ward nurses involved in the care of the tracheostomized patients.

Although the long-term mortality of 51% in our population is relatively high, but none of the patient expired during or within 24 hours of the procedure. We feel that the higher mortality rate is due to disease severity as shown by significantly higher APACHE-II score and Predicted mortality based on APACHE-II in nonsurvivor group on the day of tracheostomy. The difference of co morbidities between survivor and nonsurvivor groups was not significant. The number of days on ventilator after tracheostomy and the length of hospital stay of the patients who died were also higher in nonsurvivor group, but didn't reach statistical significance. One of the factors that may have contributed towards the mortality may be lack of intermediate care (step down or high dependency unit) and dedicated rehabilitation program in our institution. Most of the patients with tracheostomy do require intermediate care unit and long-term rehabilitation, for better outcome.

In conclusion, PCT without bronchoscopic guidance is a safe procedure with potential cost savings, which can be adopted in most of the ICUs after proper training. We recommend a tracheostomy team including an intensivist, anesthesiologist and a back up otolaryngologist, a conformation, which is present in most ICUs across United Arab Emirates.
